# Gate-readout and a 3D rectification effect for giant responsivity enhancement of asymmetric dual-grating-gate plasmonic terahertz detectors

**DOI:** 10.1515/nanoph-2023-0256

**Published:** 2023-11-09

**Authors:** Akira Satou, Takumi Negoro, Kenichi Narita, Tomotaka Hosotani, Koichi Tamura, Chao Tang, Tsung-Tse Lin, Paul-Etienne Retaux, Yuma Takida, Hiroaki Minamide, Tetsuya Suemitsu, Taiichi Otsuji

**Affiliations:** Research Institute of Electrical Communication, Tohoku University, Sendai 980-8577, Japan; School of Engineering, Tohoku University, Sendai, 980-8579, Japan; Frontier Research Institute for Interdisciplinary Sciences, Tohoku University, Sendai, 980-8578, Japan; Department of Electrical and Computer Engineering, Ecole Nationale Supérieure de l’Electronique et de ses Applications, 95000 Cergy, France; RIKEN Center for Advanced Photonics, RIKEN, Sendai, Miyagi, 980-0845, Japan; New Industry Creation Hatchery Center, Tohoku University, Sendai 980-8579, Japan

**Keywords:** terahertz detection, beyond-5G, high-electron-mobility transistor

## Abstract

We experimentally investigated the asymmetric dual-grating-gate plasmonic terahertz (THz) detector based on an InGaAs-channel high-electron-mobility transistor (HEMT) in the gate-readout configuration. Throughout the THz pulse detection measurement on the fabricated device, we discovered a new detection mechanism called the “3D rectification effect” at the positive gate bias application, which is a cooperative effect of the plasmonic nonlinearities in the channel with the diode nonlinearity in the heterobarrier between the InGaAs channel layer and the InAlAs spacer/carrier-supply/barrier layers, resulting in a giant enhancement of the detector responsivity. We also found that an undesired long-tail waveform observed on the temporal pulse photoresponse of the device is due to trapping of carriers to the donor levels in the silicon *δ*-doped carrier-supply layer when they tunnel through the barrier to the gate and can be eliminated completely by introducing the so-called inverted-HEMT structure. The internal current responsivity and noise-equivalent power are estimated to be 0.49 A/W (with the equivalent voltage responsivity of 4.9 kV/W with a high output impedance of 10 kΩ) and 196 pW/√Hz at 0.8 THz. These results pave the way towards the application of the plasmonic THz detectors to beyond-5G THz wireless communication systems.

## 1 Introduction

Beyond-5G (B5G) wireless communications, where the use of the THz bands for the wireless carrier frequencies are assumed, require highly sensitive, fast response, room-temperature operating, and on-chip terahertz (THz) detectors. Diode-based, antenna-integrated THz detectors such as Schottky barrier diodes (SBDs) [1, 2], Fermi-level-managed barrier diodes (FMBDs) [3], double-barrier resonant-tunneling diodes (RTDs) [4], triple-barrier RTDs [5], and backward diodes [6] are good candidates for this purpose. Their responsivities, however, are not sufficiently high for long-range transmissions required for B5G wireless communication networks. Transistor-based THz detectors such as single-gate Si-FETs [7, 8], GaN high-electron-mobility transistors (HEMTs) [9], or InGaAs-HEMTs [10], owing to their plasmonic and/or resistive-mixing rectification potentiality, have been investigated as an alternative candidate with ease of monolithic integration into preamplifiers and/or logic circuits. An antenna connected to the source and gate electrodes of a single-gate-type of these transistors feeds AC THz voltage between them, resulting in excitation of 2D plasmons in their channels and generation of rectified photocurrent by their hydrodynamic nonlinearities [11, 12]. Alternatively, we have developed the so-called grating-gate transistors [12–17] as a type of plasmonic THz detectors. The grating-gate structure serves as a deep-subwavelength coupler that enables direct, efficient, broadband conversion from the incident THz waves to the 2D plasmons, rather than a coupling through an integrated antenna as in the single-gate transistors. InGaAs-channel asymmetric dual-grating-gate (A-DGG) HEMTs have exhibited high internal voltage responsivities at room temperature [15–17]. Also, graphene-channel A-DGG FET [18, 19] has shown its sensitive and fast photoresponse.

Although high potentialities of the grating-gate transistors for THz detection have been demonstrated so far, there are two obstacles for further performance enhancement: (1) limited enhancement of the external responsivity by enlarging the detector active area and (2) a huge impedance mismatch with 50-Ω high-speed interconnection systems. For the application of the detector to ultrafast THz wireless communications, those critical obstacles must be resolved. Recently, we have proposed the readout of the photovoltage from the grate electrode of a A-DGG HEMT (gate-readout), instead of the conventional drain-readout, and have demonstrated that it enables the enhancement of external responsivity in proportion to the active area size as well as the impedance matching to 50-Ω interconnection systems [20].

Here, we examine the positive (forward) gate bias application to the A-DGG HEMT and demonstrate that the rectification at the heterobarrier between the InGaAs channel layer and the InAlAs spacer/carrier-supply/barrier layers due to the diode nonlinearity cooperated with the plasmonic nonlinearities, and discover a giant enhancement of the detector responsivity. First, we design and fabricate a test device based on a standard InGaAs channel heteroepitaxial layers on an InP substrate and characterize the THz detection performances, revealing an undesired long-tail waveform on its temporal photoresponse to a pulsed continuous-wave (cw) THz radiation incidence in spite of an excellent high responsivity. We investigate the cause of the long-tail response and find it is due to carrier trapping to the donor levels in the silicon *δ*-doped carrier supply layer under the forward gate-biased conditions. Second, to cope with this long-tail issue, we introduce a so-called “inverted HEMT” heteroepitaxial layered structure to relocate the carrier supply layer beneath the channel to exclude it from the through path between the channel and the gate electrode, demonstrating perfect reduction of the long tail response while preserving rather high responsivity.

## 2 Plasmonic THz detection in drain- and gate-readout configurations

**Figure 1:**
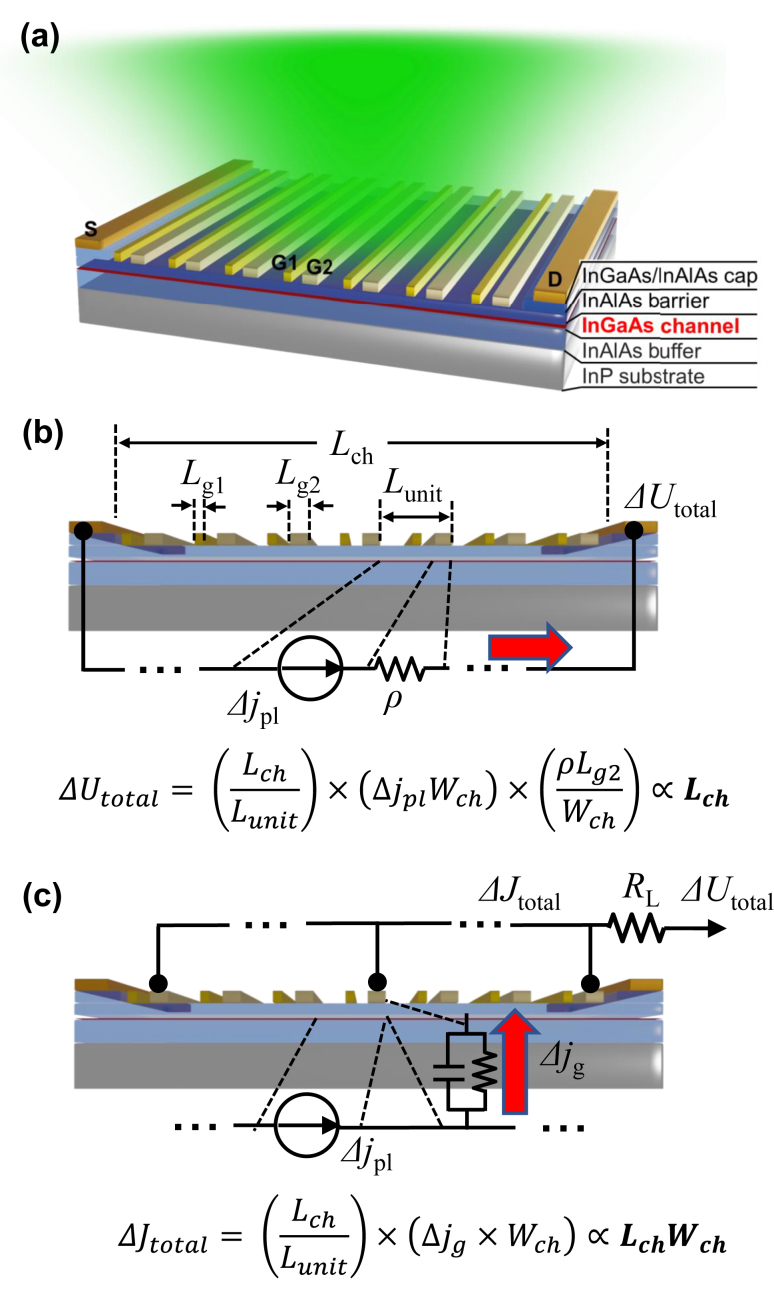
Schematic views of an InGaAs-channel A-DGG HEMT plasmonic THz detector. (a) A bird’s-eye view schematic of an InGaAs-channel A-DGG HEMT plasmonic THz detector. (b) A schematic image of the conventional plasmonic THz detection mechanism in the readout configuration of photoresponse from the drain electrode (drain-readout). (c) A schematic image of a new plasmonic THz detection mechanism, 3D rectification effect in the readout configuration of photoresponse from the gate electrode (gate-readout).

Figure 1(a) shows a schematic view of an InGaAs-channel plasmonic THz detector featured by our original A-DGG structure. It works in a depletion mode (with a negative threshold voltage of about −1 V) and has interdigitated grating-gate fingers which are placed periodically and asymmetrically. In the conventional drain-readout configuration, plasmonic cavities are formed in the “doped” channel underneath one type of grating-gate fingers, G1, by setting bias voltage to zero, while nearly-depleted “undoped” regions (i.e., voltage readout regions) are formed under the other type of grating-gate fingers, G2, by applying the bias voltage close to the threshold. The plasmonic THz detection mechanism of the A-DGG HEMT in the drain-readout configuration is described in Figure 1(a). The incident THz wave normal to the device surface with the polarization parallel to the source-to-drain direction (channel-length direction) is diffracted by the deep-subwavelength grating-gate fingers, and the diffraction waves excite the 2D plasmons in the plasmonic cavities. Then, the hydrodynamic nonlinearities of the plasmons, namely, plasmonic drag and ratchet effects [14], generate rectified DC photocurrent in the plasmon cavities. Those nonlinear effects generate unidirectional photocurrent even without source-to-drain bias due to the asymmetric gate placement [11]. The photocurrent in each unit cell flows into the adjacent voltage readout region with very high resistance, and it is converted into the photovoltage. The A-DGG arrangement causes unbalancing the photocurrent flows from/to the source to/from the drain, resulting in unidirectional rectified DC photocurrent between the source and drain electrodes [13]. Finally, the photovoltages for all the unit cells are summed up, and the output is obtained from the drain port (drain-readout configuration). Since the damping of the 2D plasmons in the InGaAs channel by electron scattering is not so small at room temperature, the plasmon excitation occurs in a non-resonant-like manner at frequencies around 1 THz and below, so that the device operates as a broadband detector at room temperature [11].

Although the internal voltage responsivity of the grating-gate plasmonic THz detectors, which is defined as a ratio of the output photovoltage to the power of the incident THz wave in the active area, is very high [15–17], a more important figure of merit of detectors for practical use is the external voltage responsivity, which is a ratio of the output photovoltage to the total power of the incident THz wave. Like other THz detectors, one way to enhance the external responsivity by increasing the coupling efficiency of the detector to the incident THz wave is the integration of the device with an external coupler, such as a Si lens [21]. Another way is to enlarge the active area size. In the drain-readout configuration, the total output photovoltage is a product of the number of grating-gate periods, photocurrent generated in each unit cell, and resistance in each depletion region (Figure 1(b)). Since the photocurrent and the resistance are proportional and inversely proportional to the channel width, respectively, they cancel each other. Then, the output photovoltage is only dependent of the channel length but is independent of the channel width. This means that enlarging the detector active area results in limited enhancement of the external responsivity. Another critical issue is the impedance mismatch with 50-Ω high-speed interconnection systems. The output impedance of the grating-gate plasmonic THz detectors is inevitably very high due to very high resistance of the depletion regions, so that the detector in the drain-readout configuration suffers from a huge impedance mismatch with the 50-Ω interconnection systems.

To overcome these obstacles, we have proposed a novel approach to use the gate electrode as a readout port (gate-readout configuration) [20]. In the gate-readout configuration (see Figure 1(c)), the photocurrent flowing through the gate electrode consists of an electron conduction current and a displacement current between the channel and each grating-gate finger. Since the grating-gate fingers are connected in parallel, the total currents in the grating-gate fingers are summed up at the gate electrode. The total photocurrent is then converted to the output voltage via an external resistance. Eventually, the output photovoltage is proportional to both the channel length and width, i.e., proportional to the active area size. Moreover, the impedance mismatch is expected to be drastically improved, as the output impedance is not limited by the series-connected resistances of the depletion regions, but is given by the parasitic series resistance of the gate electrode and the gate-to-channel capacitance which is rather low and easily meets the level of 50 Ω. Our preliminary study demonstrated those advantages experimentally [20].

## 3 Experimental

**Figure 2:**
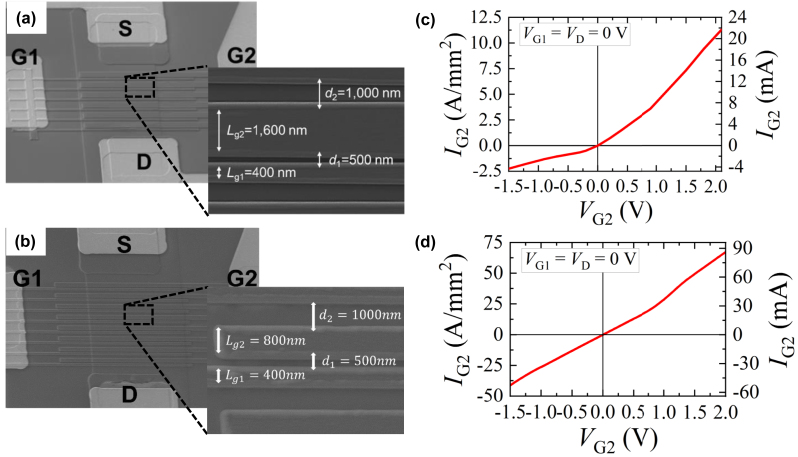
Scanning electron microscope images of (a) a normal-type and (b) an inverted-type detectors and their corresponding DC *I*_*g*2_–*V*_*g*2_ characteristics (c) and (d) (the gate 2 current density on the left axis and the total gate 2 current on the right axis).

We fabricated InGaAs-channel A-DGG HEMTs and conducted the THz pulse detection measurement on them. InP heteroepitaxial wafers were grown by molecular beam epitaxy with the layer configurations as shown in Table 1 and were processed using standard HEMT device process technologies. We prepared two types of HEMT heteroepitaxial wafers, a normal HEMT and an inverted HEMT [22] (Table 1(a) and (b), respectively). Hereafter, we shall call A-DGG HEMT plasmonic THz detectors based on those wafers as normal-type and inverted-type detectors, respectively. The main difference between them is whether the silicon-*δ*-doped carrier-supply layer for remote-doping is located above/below the channel layer in the normal/inverted HEMT. By comparing photoresponses of detectors with different structures in between the gate electrode and the channel, the physics behind the THz detection in the gate-readout configuration is expected to be revealed. The electron concentration and mobility in the In_0.53_Ga_0.47_As/In_0.7_Ga_0.3_As/In_0.53_Ga_0.47_As composite channel were 3 × 10^12^ cm^−2^ and 12,000 cm^2^/Vs, respectively, for the normal-type detector, whereas those in In_0.53_Ga_0.47_As/In_0.7_Ga_0.3_As/In_0.53_Ga_0.47_As composite channel were 2.5 × 10^12^ cm^−2^ and 9420 cm^2^/Vs, respectively, for the inverted-type detector. After device mesa isolation which defines an active area of 20 × 20 μm^2^ (for either detector), source and drain ohmic metallic contacts (Ti (20 nm)/Pt (20 nm)/Au (80 nm)/Ni (30 nm)) were formed using standard contact lithography, electron-beam evaporation, and lift-off processes. A-DGG fingers (Ti (20 nm)/Pt (20 nm)/Au (150 nm)) were formed using electron-beam lithography, electron-beam evaporation, and lift-off processes. The geometrical parameters of the A-DGG fingers were designed as follows: gate lengths *L*_*g*1_ = 400 nm and *L*_*g*2_ = 1600/800 nm (for normal-/inverted-types), gate spacings *d*_1_ = 500 nm and *d*_2_ = 1000 nm. Bird-eye view scanning-electron-microscope (SEM) images of the fabricated detectors are shown in Figure 2(a) and (b). The DC *I*_*g*2_–*V*_*g*2_ characteristics of the detectors (both the total gate 2 current and current density [total gate 2 current divided by the total area of the gate 2 fingers]) shown in Figure 2(c) and (d) were measured by a semiconductor parametric analyzer at the common-source condition with zero drain voltage. The *I*_*g*2_–*V*_*g*2_ characteristic for the normal-type detector clearly exhibits the diode-like exponential curve, whereas the characteristic for the inverted-type detector is almost linear with the current density almost one-order-of-magnitude larger than that for the normal-type detector. The latter is attributed to the very thin InAlAs barrier layer, where the tunneling barrier is so thin that the heterojunction behaves almost as an Ohmic contact.

**Table 1:** Layer designparameters of (a) normal-type and (b) inverted-type A-DGG HEMT detectors.

Layer name	Material	Thickness (nm)	Doping concentration (cm^−3^)
**(a)**			
Cap	*n*-In_0.53_Ga_0.47_As	15	1 × 10^19^
	*n*-In_0.52_Al_0.48_As	15	1 × 10^19^
Etch-stop	*i*-InP	6	–
Barrier	*i*-In_0.52_Al_0.48_As	7	–
Carrier-supply	*n*-In_0.52_Al_0.48_As	5	7 × 10^18^
Spacer	*i*-In_0.52_Al_0.48_As	3	–
Channel	*i*-In_0.53_Ga_0.47_As	3	
	*i*-In_0.8_Ga_0.2_As	8	–
	*i*-In_0.53_Ga_0.47_As	5	
Buffer	*i*-In_0.52_Al_0.48_As	100	–
Substrate	S.I.-InP	6 × 10^5^	–
**(b)**			
Cap	*n*-In_0.53_Ga_0.47_As	10	2 × 10^19^
	*n*-In_0.52_Al_0.48_As	15	7 × 10^18^
Etch-stop	*i*-InP	6	–
Barrier	*i*-In_0.52_Al_0.48_As	5	–
Channel	*i*-In_0.53_Ga_0.47_As	3	
	*i*-In_0.7_Ga_0.3_As	8	–
	*i*-In_0.53_Ga_0.47_As	3	
Spacer	*i*-In_0.52_Al_0.48_As	3	–
Carrier-supply	*n*-In_0.52_Al_0.48_As	5	7 × 10^18^
Buffer	*i*-In_0.52_Al_0.48_As	100	–
Substrate	S.I.-InP	6 × 10^5^	–

**Figure 3:**
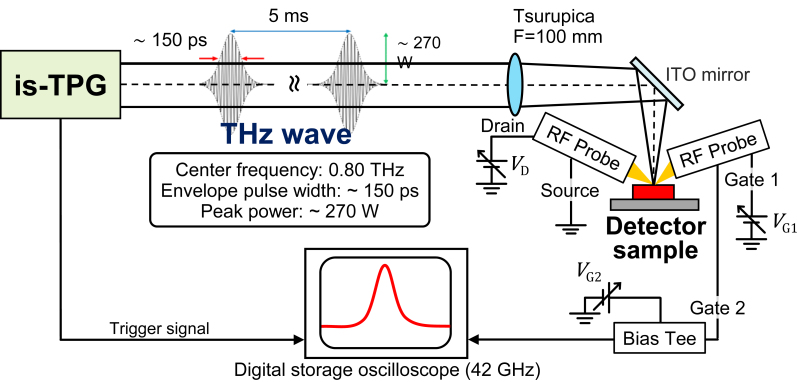
Experimental setup for pulse THz detection measurement.

We conducted THz pulse detection measurement at room temperature on the fabricated detectors using a setup shown in Figure 3. We used an injection-seeded THz-wave parametric generator (is-TPG) [23] as an ultrahigh-peak-power THz source that emits a pulsed cw THz wave with linear polarization. The center frequency of the is-TPG was set at 0.8 THz, at which the pulse width was estimated to be about 150 ps (extrapolated from the measured dependence of frequency-pulse width relation [24]) and the output peak power was about 270 W. The radiated beam was focused by a Tsurupica™ lens with the focal length of 100 mm and was redirected in the normal direction to the detector by an ITO mirror. The focused spot diameter of the beam is estimated to be about 3.67 mm. An output photovoltage signal from the gate electrode was transmitted through the 50-Ω interconnection system, and its waveform was measured by a digital storage oscilloscope.

## 4 Results and discussions

**Figure 4:**
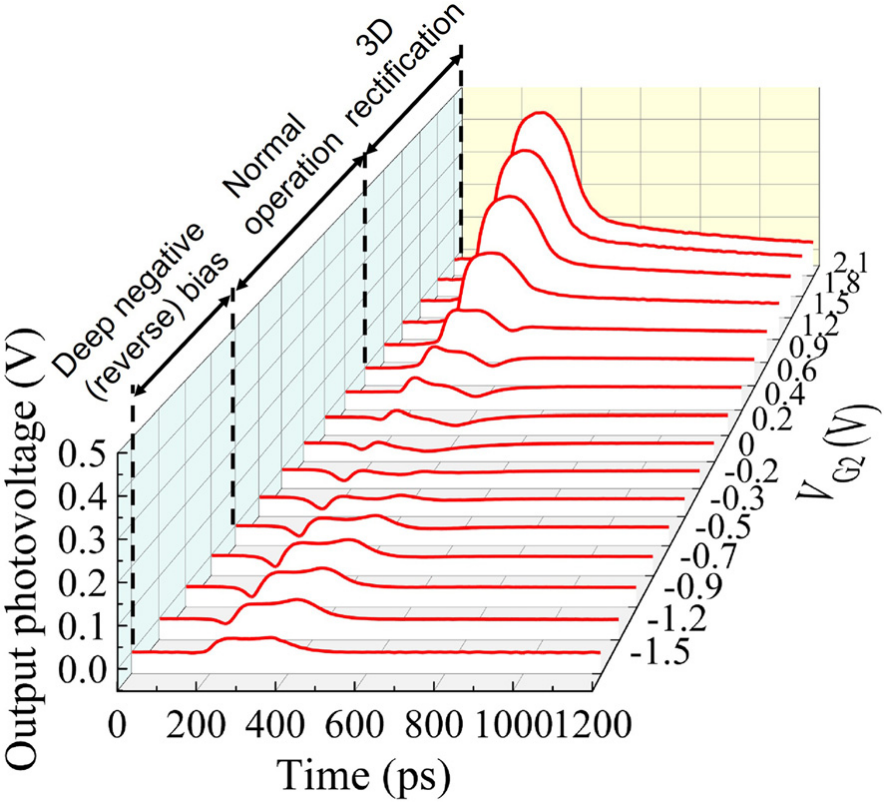
Waveforms of photoresponse from the normal-type detector with different gate bias voltages.

Figure 4 shows waveforms of output photovoltage signals from the normal-type detector with different gate bias voltages ranging from −1.5 V to +2.1 V. As can be seen, the peak photovoltage as well as the waveform drastically change with the applied gate voltage.

**Figure 5:**
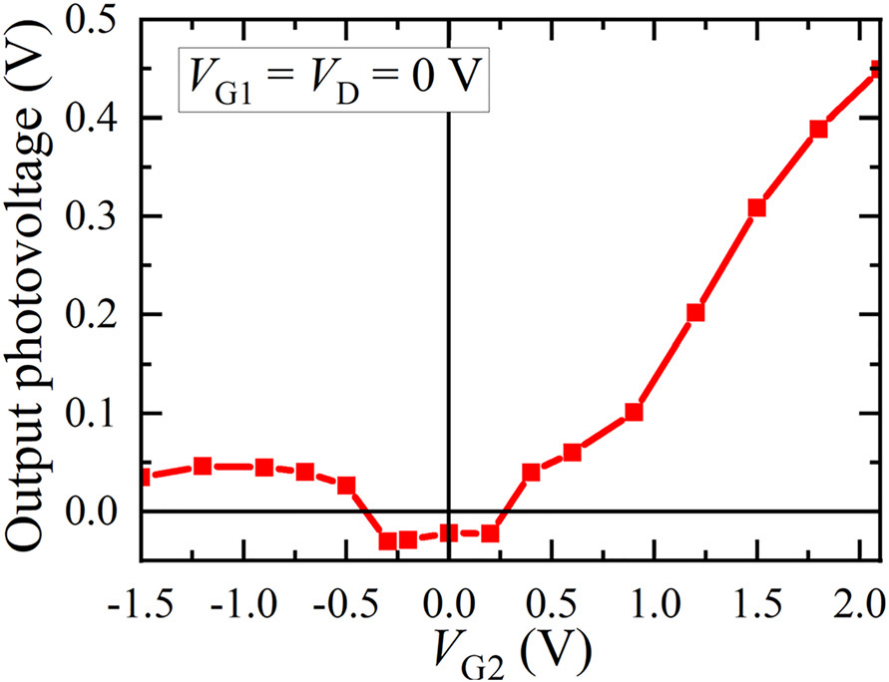
Peak photovoltage from the normal-type detector versus gate bias voltage.

Distinct shapes of the waveform allow us to categorize three operation regimes of the gate voltage: positive (forward) bias regime (+0.6 ∼ +2.1 V), normal operation regime (−0.7 ∼ +0.6 V), and deep negative (reverse) bias regime (−1.5 ∼ −0.7 V). In the positive bias regime, giant increase in the peak photovoltage is clearly demonstrated (Figure 5). The peak photovoltage is one-order-of-magnitude higher than that in the normal operation regime. The increase is almost exponential and agrees well with the *I*_*g*2_–*V*_*g*2_ characteristic in Figure 2(c). This indicates that the observed photovoltage enhancement is associated with the diode current nonlinearity at the heterobarrier formed between the InGaAs channel layer and the InAlAs spacer/carrier-supply/barrier layers.

**Figure 6:**
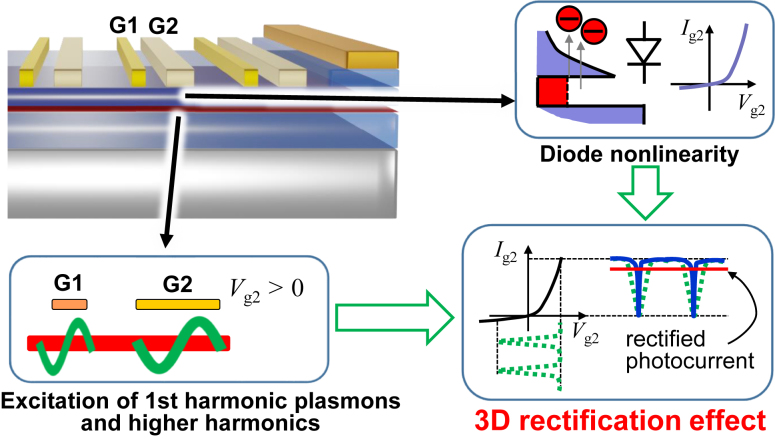
A Schematic image of a new plasmonic THz detection mechanism, 3D rectification effect in the readout configuration of photoresponse from the gate electrode (gate-readout).

The mechanism of the giant photovoltage enhancement can be explained as follows. At a high positive gate bias voltage, the thickness of the heterobarrier becomes sufficiently thin, so that the electrons in the channel can tunnel the heterobarrier (Figure 6). This provides the exponentially increasing diode current nonlinearity, which is similar to the resonant-tunneling nonlinearity in a single-gate resonant-tunneling transistor detector proposed theoretically in Ref. [25]. Then, the harmonically oscillating 2D plasmonic photocurrent as well as all the higher harmonics are rectified by the diode current nonlinearity. This results in multiplication of the diode nonlinearity (vertical direction to the channel) and the plasmonic nonlinearity (in-plane direction to the channel), leading to the giant “3D rectification effect”. These physical descriptions are supported qualitatively by the following simplest formulation. First of all, the output voltage signal (i.e., the potential variation) generated by the plasmonic hydrodynamic nonlinearity in response to an input voltage signal (i.e., harmonically oscillating potential of the 2D plasmons excited by the incident THz waves), *δV*cos*ωt*, can be represented by a nonlinear response function, *F*:

(1) $$\begin{array}{ll} F\left(\delta V\cos\omega t\right) & =F^{\prime \prime }\left(0\right){\left(\frac{\delta V}{2}\right)}^{2}+F^{\prime }\left(0\right)\delta V\cos\omega t\\ & \;+F^{\prime \prime }\left(0\right){\left(\frac{\delta V}{2}\right)}^{2}\cos2\omega t+\cdots \;. \end{array}$$

Then, by expressing the nonlinear diode current as *G* = exp(*eV*/*ηk*_*B*_ *T*_*e*_)−1, where *η* is the ideality factor of the diode nonlinearity, *k*_*B*_ is the Boltzmann constant, *T*_*e*_ is the electron temperature, *e* is the elementary charge, the photocurrent from the gate electrode can be derived as follows:

(2) $$\begin{array}{ll} & \exp\left\{\frac{e}{\eta k_{B}T_{e}}\left[V_{g}-F\left(\delta V\cos\omega t\right)\right]\right\}-1\\ & \;\approx \exp\left(\frac{eV_{g}}{\eta k_{B}T_{e}}\right)\exp\left\{-\frac{e}{\eta k_{B}T_{e}}\left[F^{\prime \prime }\left(0\right){\left(\frac{\delta V}{2}\right)}^{2}\right.\right.\\ & \;+\left.\left.F^{\prime }\left(0\right)\delta V\cos\omega t+F^{\prime \prime }\left(0\right){\left(\frac{\delta V}{2}\right)}^{2}\cos2\omega t+\cdots \;\right]\right\}. \end{array}$$

Note that the gate bias voltage is superposed onto the potential variation, *F*(*δV*cos*ωt*), in the channel. It is apparent from Eq. (2) that the harmonically oscillating 2D plasmonic photocurrent as well as all the higher harmonics are rectified by the diode current nonlinearity (as in the second factor) and that the plasmonic nonlinearity is multiplied by the exponential diode nonlinearity. The first exponential factor in Eq. (2), which corresponds to the nonlinear diode *I*–*V* characteristic measured in Figure 2(b), is strong evidence of our claim that the dominant effect in the enhancement of the giant photovoltage observed in Figure 5 originates from the heterobarrier diode nonlinearity. The multiplication factor in the ideal case, where the ideality factor is *η* = 1, would reach more than 44 at *V*_*g*_ = +0.1 V and at room temperature, *T*_*e*_ = 300 K. Although that of the fabricated normal-type A-DGG HEMT, where the ideality factor extracted from the fitting of the *I*–*V* characteristic in Figure 2(c) is *η* is about 100 at *V*_*g*_ = +2.1 V, it can be much improved by an appropriate heterobarrier design (see the discussion in Section 5). More strict theoretical analysis needs a self-consistent numerical model that couples electron transport equations (in the channel and between the channel and gate) with the Maxwell equations and will be a future work.

**Figure 7:**
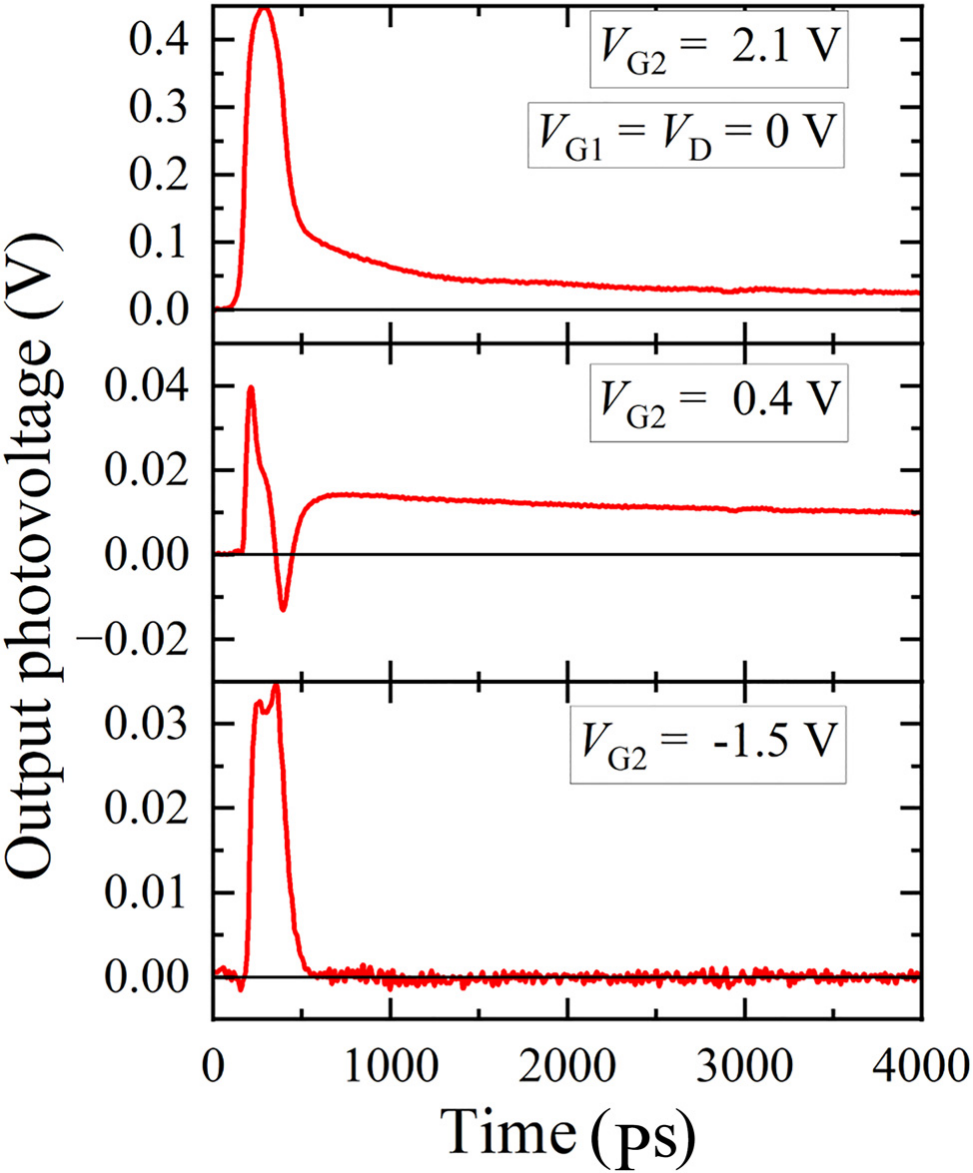
Waveforms of the photoresponse from the normal-type detector at gate bias voltages in different operation regimes.

**Figure 8:**
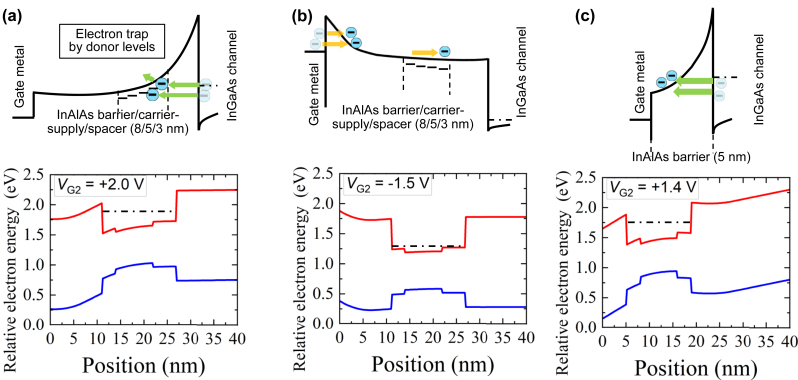
Band diagrams of the detector under the gate 2 electrode at gate bias voltages calculated by a semiconductor device simulator APSYS and zoom-in schematic views of their conduction band diagrams corresponding to (a) 3D rectification regime and (b) deep negative bias regime for the normal-type detector and (c) for the inverted-type detector.

At the same time, the photoresponse waveform in the positive (forward) bias regime (the top panel in Figure 7) consists not only of the main photovoltage Gaussian-like pulse, corresponding to the input THz pulse, but of a long tail waveform following the main pulse. The characteristic decay time of the long tail extracted by fitting to an exponential function, exp(−*t*/*t*_decay_), was *t*_decay_ = 13 ns. Oppositely, no tail in the deep negative bias regime was observed (the bottom panel in Figure 7). Considering the band structures with the positive (forward) and negative (reverse) gate bias applications depicted in Figure 8(a) and (b), we claim that the origin of the long tail is attributed to the carrier trapping at the donor levels in the InAlAs carrier-supply layer when electrons tunnel from the InGaAs channel to the gate electrode. As a result of the remote doping from the silicon-*δ*-doped carrier supply layer to the channel layer to form the 2D electron gas, the high-concentration ionized donor levels (7 × 10^18^ cm^−3^) slightly below the conduction band edge are remained in the carrier-supply layer. Under a positive (forward) gate bias voltage application, electrons in the channel can tunnel either to the conduction band in the carrier-supply layer directly or to those donor levels. In the latter case, electrons are trapped for a long time and should cause the long tail waveform.

Besides, in the normal operation regime, the waveform is in the form of a superposition of a Gaussian pulse and its derivative, together with the long tail (the middle panel in Figure 7). This derivative waveform originates from the capacitive coupling, i.e., displacement current, between the gate fingers and the channel, which is proportional to dΔ*Q*_ph_ */*d*t* = *C*_*g*_ dΔ*V*_ph_ */*d*t*, where Δ*Q*_ph_ is the charge accumulated by the nonlinear photocurrent rectification, *C*_*g*_ is the gate-channel capacitance, and Δ*V*_ph_ is the photovoltage.

**Figure 9:**
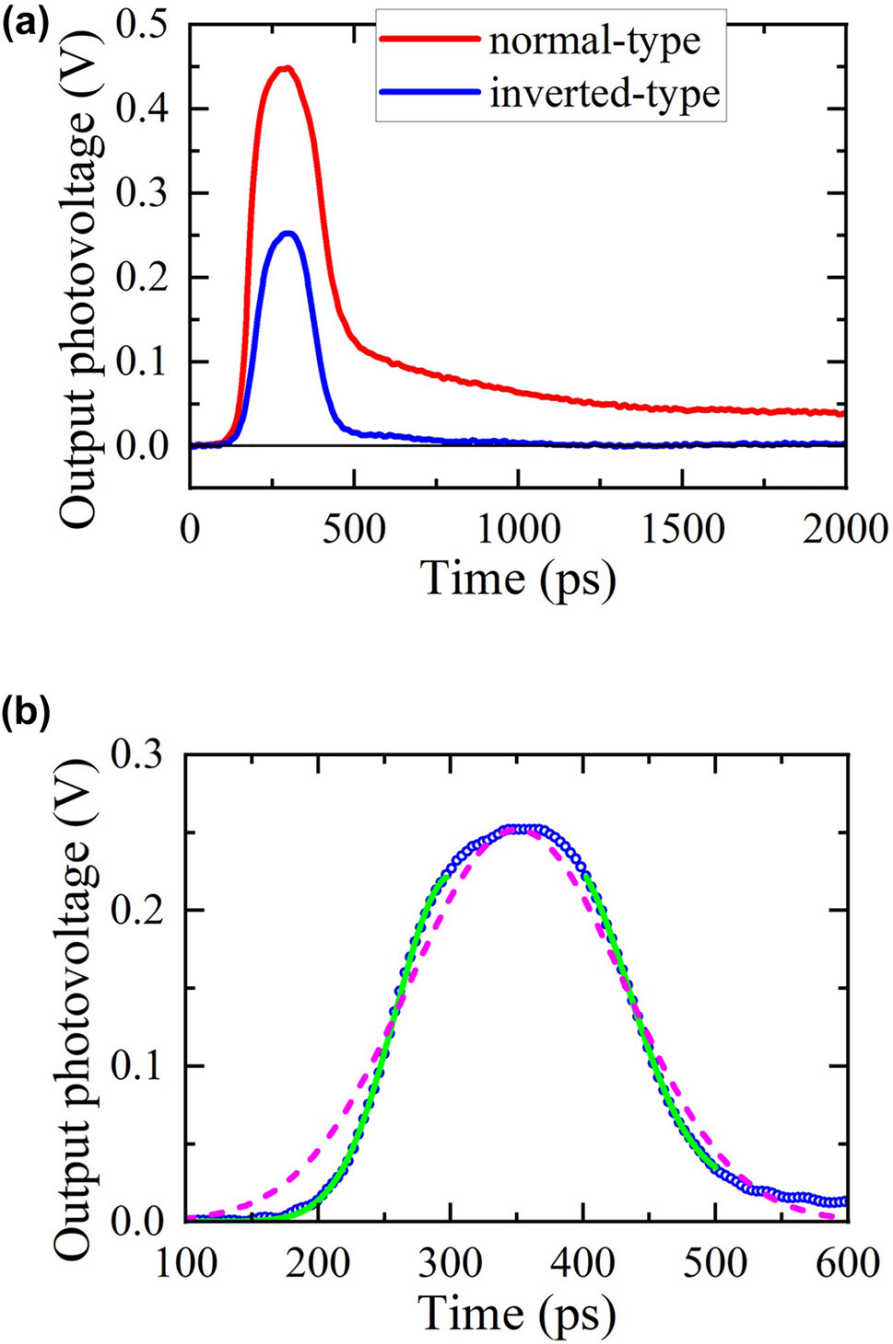
Photoresponses of the normal-type detector and the inverted-type detector. (a) Measured waveforms of photoresponses from the normal-type detector and the inverted-type detector. (b) Enlarged view of the measured waveform for the inverted-type detector (blue open circles) with a full Gaussian fitting curve (magenta dashed line) and partial Gaussian fitting curves only near the rise/fall edges (green solid lines).

Next, we compare the photoresponses of the inverted-type detector with that of the normal-type detector. As shown in Table 1(b), the carrier supply layer in the inverted-type detector is placed beneath the channel to exclude it from the through path between the channel and the gate electrode. Figure 9 shows the comparison of the output photoresponses from those in the normal-type detector. As clearly seen, the tail photoresponse waveform completely disappeared for the inverted-type detector. In contrast to the normal-type detector, the through path between the channel layer and the gate electrode in the inverted-type detector is occupied only by the undoped InAlAs barrier layer (Figure 8(c)). Then, the electrons in the InGaAs channel layer directly tunnel into the conduction band in the barrier layer without any undesired trapping, which results in the fast photoresponse without any trapping. This result evidently confirms that the donor levels in the InAlAs carrier-supply layer are the cause of the long tail waveform observed in the normal-type detector and simultaneously demonstrates that it was eliminated completely by introducing the inverted-HEMT structure. The lower peak photovoltage for the inverted-type detector compared with the normal-HEMT detector is mainly due to the lower diode nonlinearity as manifested in the DC *I*_*G*2_–*V*_*G*2_ characteristic of the inverted-type detector in Figure 2(d). Currently prepared heteroepitaxial layer design of the inverted-type HEMT is a standard for normal transistor operation so that the InAlAs barrier layer is very thin, resulting in Ohmic-like linear current-voltage characteristics under the negative gate bias conditions. It should be much improved by an appropriate design with a thicker barrier layer for the purpose of plasmonic THz detection. The full-width at half-maximum (FWHM) of the output pulse for the inverted-type detector was ∼190 ps, whereas that of the input THz pulse is about 150 ps. Considering that the rise and fall times are each dulled by 20 ps, the response time of the inverted-type detector is estimated to be on the order of 10 ps. Therefore, the inverted HEMT structure with an appropriate barrier layer design is a suitable structure for high-speed THz wireless communications. On the other hand, the appearance of the long tail waveform for the normal-type detector could be utilized as an intrinsic smoothing mechanism of THz pulses for imaging and sensing applications, if fast response of a THz detector is not a prerequisite.

To see a deeper insight, it should be noted that the output pulse waveform does not well fit the Gaussian function with the FWHM of 190 ps, as shown in Figure 9(b), and has two distinct features: (1) asymmetric rise and fall times, which excellently fit Gaussian functions with FWHMs of 101 and 114 ps, respectively, shorter than that of the incident THz pulse, and (2) a small, short tail on the order of 100 ps. The first feature was also observed for the commercial SBD detector and can be explained as the nonlinear pulse compression near the saturation regime at a very high input power [24]. The second feature could be associated with combination of unintentional factors such as reflected input THz pulse by the back surface of the InP substrate, the displacement photocurrent by the capacitive coupling of the gate fingers and the channel, and nontrivial distributed impedance formed by the gate fingers and the channel as an output waveguide along the gate-width direction. Further quantitative investigation on the pulse photoresponse is necessary as a future work.

## 5 Estimation of internal current responsivity and NEP

As stated in Ref. [3], the current responsivity is an important figure of merit of THz detectors. First, we characterized the internal current responsivity of the normal-type detector. Considering the THz power generated from the is-TPG and losses by a Tsurupica™ lens and an ITO mirror (∼5 % for each at 0.8 THz), the incident THz power upon the detector surface is estimated to be 243 W. The focal length of the Tsurupica™ lens, 100 mm, together with the collimated beam diameter of the THz radiation, 13 mm, give the spot diameter approximately 3.67 mm of the THz radiation on the detector active area. The power irradiated onto the active area, which has the size 20 × 20 μm^2^, is then calculated to be 18.3 mW. The peak photovoltage for the normal-type detector at *V*_*g*2_ = +2.1 V is 0.449 V, as shown in Figure 9(a). Then, the internal voltage responsivity is 24.5 V/W. Finally, considering the current-to-voltage conversion at the 50-Ω load resistance, the internal current responsivity is estimated to be 0.49 A/W. To benchmark the responsivity with existing detectors by assuming a high output load impedance, say 10 kΩ, the voltage responsivity is estimated to be 4.9 kV/W, which is by an order of magnitude higher than the record performance reported in the drain-readout-type plasmonic A-DGG HEMT detectors.

**Figure 10:**
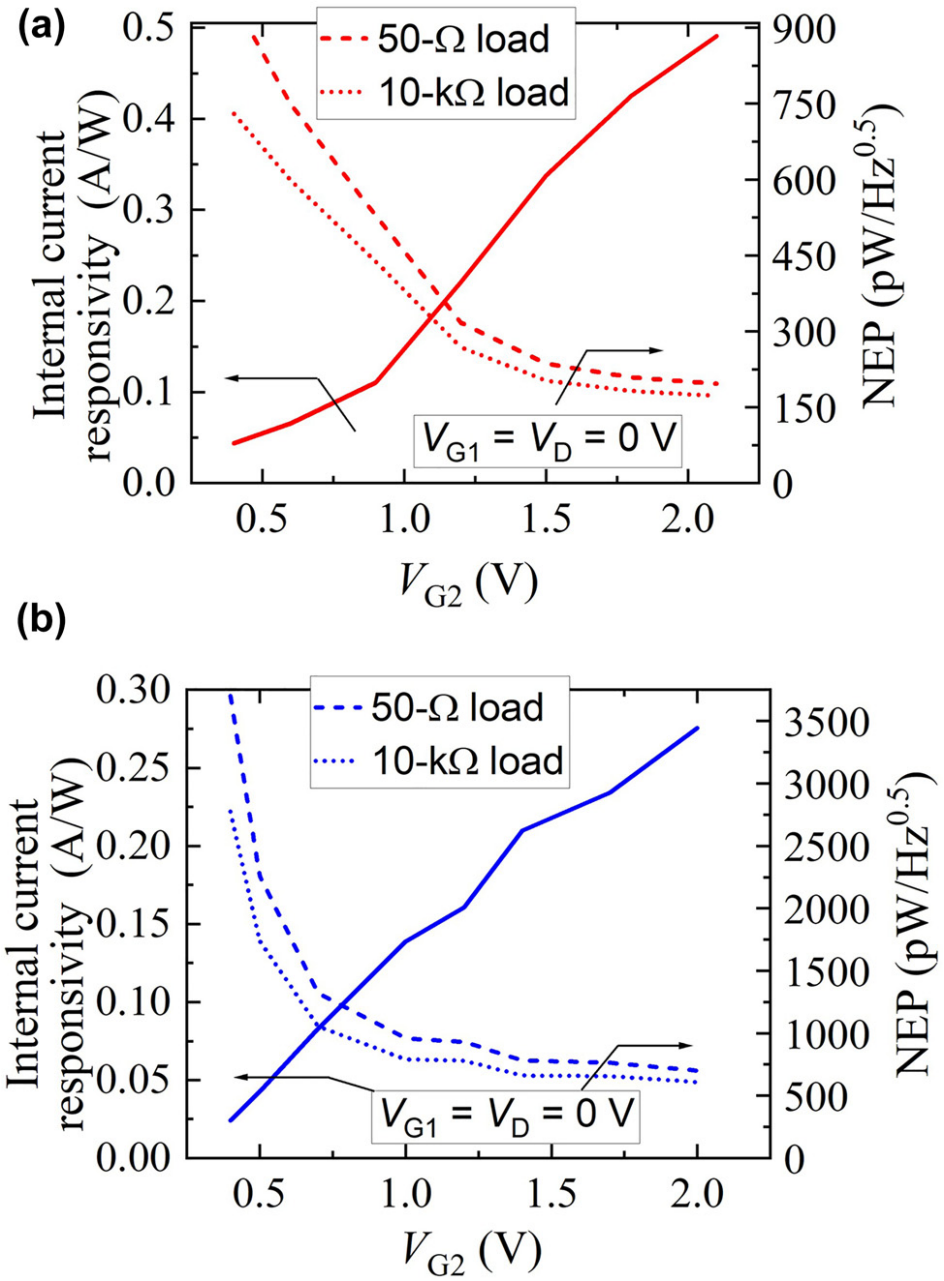
Internal current responsivity and NEP of (a) the normal-type and (b) inverted-type A-DGG detectors as functions of the gate 2 voltage with different output load impedances.

The internal noise equivalent power (NEP) of our detector taking into account both the thermal noise and the shot noise is estimated as follows:

(3) $$\text{NEP}=\frac{N_{\text{th}}+N_{\text{sh}}}{R_{i}}=\frac{\sqrt{4k_{B}T_{e}/R_{g2}}+\sqrt{2eI_{g2}}}{R_{i}},$$

where *R*_*i*_ is the internal current responsivity, *N*_th_ and *N*_sh_ are the thermal noise and shot noise factors (in the unit of A/√Hz), respectively, *I*_*g*2_ is the total gate 2 current, and *R*_*g*2_ is the gate-to-channel resistance. The generation-recombination (GR) noise associated with the electron trapping in the normal-type detector might become an unavoidable noise source depending on the density of trapped electrons at the doped-impurity-originated centers, whereas it should be negligibly small for the inverted-type detector. Nevertheless, the ever-reported data for the III–V heterojunctions with doped layer(s) show the GR rate of electrons predominantly stay ranging 1–100 MHz [26]. Although the flicker noise (1/*f* noise) in a semiconductor detector can be a nonnegligible noise source at very low frequencies usually ranging 1–10 kHz [27], use of such a low frequency region is usually avoided in data signals for high-speed wireless communication systems, and low-frequency noise should be dropped off by inserting high-pass filter(s) in transmission lines. In fact, the bias tee used in our measurement setup (Figure 3) has a role of a high-pass filter for the signal component and has the cutoff frequency of 50 kHz, so that the flicker noise generated from the detector could be dropped off. The internal NEP and internal current responsivity as functions of the gate 2 voltage are shown in Figure 10(a) with different output load impedances (50 Ω and 10 kΩ). As seen, the minimum NEP is 196 pW/√Hz for 50-Ω load and 172 pW/√Hz for 10-kΩ load. The internal NEPs for different load impedances do show little difference because they are dominated by the shot noise, which does not depend on the load impedance.

Second, the internal current responsivity and NEP for the inverted-type detector was characterized as shown in Figure 10(b), where the maximum responsivity is 0.27 A/W (2.7 kV/W) and minimum NEP is 700 pW/√Hz (608 pW/√Hz) for 50-Ω (10-kΩ) load. The difference between the normal- and the inverted-type detectors is mainly due to the difference between their *I*_*g*2_–*V*_*g*2_ characteristics. As shown in Figure 2(c) and (d), the normal-type detector clearly exhibits the diode-like exponential characteristic, whereas the inverted-type detector shows less-nonlinear, superlinear characteristic. The linear behavior of the current density as well as its huge absolute value (6 times higher than that for the normal type) is mainly caused by the very thin InAlAs barrier layer (5 nm) of the heteroepitaxial wafer designed for general ultrahigh-frequency transistor amplifiers, where the tunneling barrier is so thin that the heterojunction behaves almost as an Ohmic contact. In total, the nonlinear rectification effect is stronger for the normal-type detector than for the inverted-type detector, resulting in about twice larger internal current responsivity for the normal-type detector than that for the inverted type. The difference in the NEPs between the inverted-type and the normal-type is much larger (about 4 times) than that in their responsivities because of the larger shot noise power predominated by the 4-times higher total gate 2 current for the inverted type than for the normal type as shown in Figure 2(c) and (d). The aforementioned lower performance of the fabricated inverted-type detector is due to the heteroepitaxial layers structure designed not for high-responsivity but for ultrahigh-frequency operation performances. With appropriate choice of the barrier layer design (material and thickness), further enhancement of the detection performance for the inverted-type detector is highly feasible.

Now we compare the internal NEP and internal current responsivity of our detector with those of other THz detectors measured at similar frequencies. The external current responsivity of a Si-FET (at zero drain bias voltage) is reported to be 0.045 A/W at 0.8 THz, with the NEP is 48 ∼ 70 pW/√Hz in the range 0.6 ∼ 1.5 THz [8]. The internal voltage responsivity and the NEP of an A-DGG HEMT detector at 1 THz under zero-drain-bias, depleted-channel condition in the drain-readout configuration [16] were reported to be 2.2 kV/W and 15 pW/√Hz. With the channel resistance on the order of 100 kΩ, the internal current responsivity of the normal-type detector in the drain-readout configuration is estimated to be on the order of 0.01 A/W. Thus, it is demonstrated that the A-DGG HEMT detector in the gate-readout configuration surpasses the existing plasmonic THz detectors in the drain-readout configuration in terms of the internal current responsivity, which in turn demonstrates its superior performance for the high-speed wireless communications. The external current responsivity of an FMBD in the zero-bias condition was reported to be 0.3 ∼ 0.4 A/W at 0.8 THz [3]. The external current responsivity and the NEP of an RTD in the normal square-low detection mode were 0.1 A/W and 340 pW/√Hz at 0.78 THz [4]. Compared with those conventional THz detectors, the internal current responsivity of the normal-type detector in the gate-readout configuration is higher. This confirms that our detector potentially surpasses other types of high-sensitivity, high-speed semiconductor THz detectors. The larger internal NEP of our detector than the conventional THz detectors is due to the large DC gate current that causes the large shot noise and is an issue to be resolved as a future work. Since the current device design of our detector is inherited from usual InGaAs-channel HEMTs for normal transistor operation that does not care for the gate-channel forward biasing conditions under consideration. The occurrence of the nonlinear 3D rectification requires a relatively large positive gate bias voltage, which inevitably accompanies with the large DC gate current in general structural design for standard HEMTs no matter which the HEMT is normal- or inverted-type, suffering from large NEPs. In order to optimize the HEMT design so as to minimize the NEPs for use in THz radiation detection, an appropriate gate-channel heterobarrier design that maximize the nonlinear 3D rectification effect under a low or even zero gate bias voltage with reduced gate-channel current is needed, promising a drastic improvement of the NEP as well as further enhancement of the responsivity. Such an improved heterobarrier design will particularly be effective for the invert-HEMT structure.

Instead of the direct (square-low) detection that has been discussed so far, the heterodyne detection is well-known to effectively enhance the current responsivity and NEP. In the heterodyne detection, the frequency of the input signal (the THz radiation incidence in our case) is mixed down to a much lower intermediate frequency by nonlinear mixing with a local oscillator signal. The external current responsivity and the NEP of an RTD in the heterodyne detection mode, where a local oscillator signal is generated inside the RTD by its negative dynamic conductance, were reported to be 3.0 A/W and 7.7 pW/√Hz at 0.78 THz [4], which are more than one-order-of-magnitude better than those in the square-low detection mode, 0.1 A/W and 340 pW/√Hz. By introducing an external THz local oscillator, our detector is also capable of operating in the heterodyne detection mode, so that further performance enhancement is expected.

It is also worth mentioning about the matching of output impedance of our detector to 50-Ω interconnection systems, which is an important condition for high-fidelity, high-speed signal transmission to suppress waveform distortion. Like any other detectors, if the internal impedance of the detector is larger or smaller than 50 Ω, a series or shunt matching resistance must be inserted to meet the 50-Ω near-end impedance condition. In the case of the normal-type A-DGG HEMT with its active area of 20 × 20 μm^2^, the output resistance is determined by the gate-to-channel resistance and was about 100 Ω at *V*_*g*2_ = +2.1 V and *V*_*g*1_ = *V*_*d*_ = 0 V (see Figure 2(c)), where the entire channel is highly electron-doped and its resistance is on the order of 100 Ω, and the impedance matching can be accomplished by inserting the same amount of a shunt resistance. Although we directly connected the detectors to a 50-Ω interconnection system in the measurement setup without such a sending-end impedance-matching circuit, a notable distortion was not observed because the far-end impedance was matched to 50 Ω to well suppress multiple reflections between the near and far ends. The experimental results confirmed that the relatively low-impedance nature of gate readout configurations does not need to be sensitively concerned with near-end impedance matching. This is a strong merit for the gate-readout configuration. In contrast, this is not the case for the standard drain-readout configuration. In the drain-readout configuration, one of the gate voltages, *V*_*g*2_, is set to be close to a threshold voltage, so that the channel regions underneath the gate fingers are almost depleted and the total channel resistance is inevitably very high (see the description of the operation principle in the drain-readout configuration at the beginning of Section 2). This in turn causes very huge output resistance typically on the order of 100 kΩ as in Ref. [15]. From this comparison, it is evidently shown that the gate-readout configuration is superior to the conventional drain-readout configuration regarding also to the impedance matching to 50-Ω interconnection systems. Also, such a huge impedance mismatching in the drain-readout configuration causes a huge drop of the output photovoltage when the detector is connected to 50-Ω interconnection systems, although the photovoltage generated inside the detector is large. In the gate-readout configuration, such a drop does not arise due to its low-impedance nature, resulting in the output photovoltage markedly higher than that in the drain-readout configuration.

## 6 Conclusions

We fabricated the asymmetric dual-grating-gate plasmonic THz detectors based on InGaAs-channel HEMTs and conducted the THz pulse detection measurement in the gate-readout configuration. At the positive gate bias application, which corresponds to the forward bias application to the gate-channel diode, a giant enhancement of the output peak photovoltage from the detector, which is one-order-of-magnitude higher than that under the negative gate bias conditions, was observed. From the diode-like exponential gate current-voltage characteristic, we identified that the giant enhancement is originated from a new detection mechanism named the “3D rectification effect”, which is a cooperative effect of the plasmonic nonlinearities in the channel with the diode nonlinearity in the heterobarrier between the InGaAs channel layer and the InAlAs spacer/carrier-supply/barrier layers. We found that an undesired long-tail waveform with the decay time of 13 ns appearing in the temporal pulse photoresponse is due to trapping of carriers to the donor levels in the silicon *δ*-doped carrier-supply layer when they tunnel from the channel to the gate through the barrier. By introducing the so-called inverted-HEMT structure, where the carrier supply layer is relocated beneath the channel, we succeeded in eliminating the long-tail waveform completely. In addition, the response time of the fabricated inverted-type detector was shown to be on the order of 10 ps. The internal current responsivity and noise-equivalent power of the fabricated normal-type (inverted-type) detector were estimated to be 0.49 A/W (0.27 A/W) and 196 pW/√Hz (700 pW/√Hz) at 0.8 THz (with the equivalent voltage responsivity and NEP of 4.9 kV/W (2.7 kV/W) and 172 pW/√Hz (608 pW/√Hz) with a high output impedance of 10 kΩ), exceeding the performances of existing THz detectors in terms of the internal current responsivity. Also, the comparison of the output resistance of the detector in the gate-readout configuration, about 100 Ω, with that in the conventional drain-readout configuration, typically on the order of 100 kΩ, showed that the gate-readout configuration is markedly superior to the drain-readout configuration in terms of impedance matching to 50-Ω interconnection systems, which greatly suppresses distortion of the output waveform as well as drop of the output photovoltage. These results pave the way towards the application of the plasmonic THz detectors to beyond-5G THz wireless communication systems.
